# GPR109A controls neutrophil extracellular traps formation and improve early sepsis by regulating ROS/PAD4/Cit-H3 signal axis

**DOI:** 10.1186/s40164-023-00376-4

**Published:** 2023-01-31

**Authors:** Wenjin Guo, Qian Gong, Xiaofeng Zong, Dianjun Wu, Yuhang Li, Huijie Xiao, Jie Song, Sheng Zhang, Shoupeng Fu, Zhichun Feng, Lu Zhuang

**Affiliations:** 1 College of Veterinary Medicine, Jilin University, Changchun, 130062 China; 2 Senior Department of Pediatrics, The Seventh Medical Center of PLA General Hospital, Beijing, China; 3 Institute of Pediatrics, The Seventh Medical Center of PLA General Hospital, Beijing, China; 4 National Engineering Laboratory for Birth Defects Prevention and Control of Key Technology, Beijing, China; 5 Beijing Key Laboratory of Pediatric Organ Failure, Beijing, China; 6 Department of Gastrointestinal and Colorectal Surgery, China-Japan Union, Hospital of Jilin University, Changchun, 130033 China; 7 College of Animal Sciences, Zhejiang University, Hangzhou, 310030 China

**Keywords:** Sepsis, Neutrophil extracellular traps, GPR109A, Cecum ligation and puncture, Cit-H3

## Abstract

**Background:**

Neutrophil extracellular traps (NETs) is the key means for neutrophils to resist bacterial invasion. Sepsis is a systemic inflammatory response syndrome caused by infection.

**Methods:**

In our study, qRT-PCR was used to detect the gene expression in neutrophils, Western blot was used to detect the protein expression in mouse tissues and neutrophils, flow cytometry was used to detect the purity of neutrophils in the whole blood and immunofluorescence was used to detect the NETs formation.

**Results:**

In this study, we analyzed the NETs formation in the blood of patients with sepsis. The results showed that a large number of NETs appeared. And the expression of GPR109A in neutrophils of patients with sepsis was significantly up regulated. Then we collected neutrophils from WT mice and GPR109A^−/−^ mice and found that GPR109A knockout could significantly inhibit the early NETs formation of neutrophils. The results also showed that knockout of GPR109A or inhibition of the NETs formation could increase the inflammatory response of liver, spleen, lung and kidney in mice, thus affecting the disease process of sepsis. Then we observed the death of mice in 16 days. The results showed that inhibiting the NETs formation could significantly affect the early mortality of mice, while knocking out GPR109A could directly affect the mortality of the whole period.

**Conclusions:**

This study confirmed the regulatory effect of GPR109A on early NETs formation for the first time, and provided a new target for the treatment of sepsis.

**Supplementary Information:**

The online version contains supplementary material available at https://doi.org/10.1186/s40164-023-00376-4.

## Background

Sepsis refers to a systemic infection that occurs when pathogenic bacteria entering the blood circulation via various mechanisms, growing and reproduce in the blood, and produce a large number of toxins [1]. Studies have shown that sepsis is mainly represents the antagonism between the pathogen and the host immune system [2, 3]. Generally, severe bacterial infection is the main cause of sepsis [4]. At present, the treatment of sepsis is mainly based on antibiotics, but the extensive use of antibiotics leads to bacterial resistance, which affects follow-up treatment [5–7]. Therefore, developing a new antimicrobial target from the perspective of innate immunity is of great significance for the clinical treatment of sepsis [8]. Among many natural immune barriers, neutrophils are one of the main immune cell populations that functions in the early response to bacterial invasion, and they play an important role in the early stage of sepsis [9]. Neutrophils mainly play a bactericidal role via the formation of neutrophil extracellular traps (NETs) [10]. Improving NETs formation in the early stage of sepsis is an important means of inhibiting the circulation of bacteria in the blood.

Neutrophils are the main natural immune barrier against pathogenic microorganisms [11]. After being stimulated by external pathogens, neutrophils will form NETs, which can eliminate pathogens [12]. Therefore, in the early stage of many bacterial diseases, NETs can kill pathogens in a timely and rapid manner. NETs are important immune barriers by which the body to prevents the invasion of external pathogens [13]. Recent studies have shown that NETs are a natural defense barrier composed of neutrophil elastase (NE), myeloperoxidase (MPO) and histones, and NETS are formed by neutrophils after stimulation with external factors, such as bacteria, lipopolysaccharide (LPS), and phorbol myristate (PMA) [11]. Many studies have shown that NETs can capture and kill pathogenic microorganisms, and inhibit the growth and reproduction of pathogenic bacteria in vivo [14]. Some studies have found that the release of NETs may also be related to tissue and organ damage [15, 16]. However, we know that any function is a double-edged sword. The body needs to regulate the formation of NETs at any time to ensure that NETs kill pathogens without causing excessive damage to other tissues and organs. Current studies have shown that NETs formation is mainly related to reactive oxygen species (ROS), PAD4 and histone citrullination, but the specific mechanism is not clear [17]. Therefore, further studies on the mechanism of NETs formation are of great significance for the clinical treatment of sepsis.

G protein-coupled receptors can bind chemicals in the environment surrounding cells and activate a series of signaling pathways in cells, which eventually leads to changes in the cell state [18]. However, there are relatively few studies on the relationship between G protein-coupled receptors and NETs formation. In my previous study, the GPR109A and Cit-H3 levels in neutrophils from patients with bacterial sepsis were significantly increased, and neutrophils in septic patients spontaneously formed NETs in vitro. These results suggested that GPR109A may play an important role in bacterial sepsis through NETs formation. GPR109A belongs to the G protein-coupled receptor family [19]. Recently, some studies have shown that GPR109A plays an important role in inflammatory diseases such as sepsis and nephrosis [20, 21]. GPR109A knockout aggravates the inflammatory response, increase the levels of proinflammatory cytokines, and reduce the survival rate of mice [22, 23]. This suggests that GPR109A may play a role in protecting human health. However, the mechanism underlying GPR109A-mediated protection of septic mice is unclear, so we studied the mechanism by which GPR109A functions in the formation of NETs and its effect on sepsis.

## Methods

### Isolation of human neutrophils

This study was carried out in accordance with the recommendations of the Declaration of Helsinki guidelines and approved by the ethics committee of the China-Japan Union Hospital of Jilin University (20201115). Informed consent was obtained from all patients and volunteers involved in this study. The patients with septicemia are men aged 28–35 years old, who are all acute bacterial infections. The age of healthy volunteers was also between 28 and 35 years old, and they had no other medical history. Blood samples from healthy volunteers and patients with sepsis were collected by doctors, and then neutrophils in blood were separated for follow-up experiment. For specific separation steps, refer to the kit instructions (LZS11131, tbdscience, China). Neutrophils isolated from the blood of healthy volunteers and patients with sepsis were cultured in human peripheral blood neutrophil medium.

### Animals and experimental design

The GPR109A^−/−^ mice(C57BL/6) was a generous gift from Dr. Martin Sager (Zentrale Einrichtung für Tierforschung und Tierschutzaufgaben der Heinrich-Heine Universität Düsseldorf, Germany). The experiments were approved by the Jilin University Institutional Animal Care and Use Committee. Male WT and GPR109A^−/−^ mice at the age of 8 weeks were used for experiments. Mice were kept under a 12-h night-day rhythm with free access to water and food. Cecum ligation and puncture (CLP) model was constructed according to the operation steps of Daniel Rittirsch. WT mice were randomly divided into NT group, sham operation group, CLP group and CLP + DNase I group. GPR109A^−/−^ mice were randomly divided into NT group, sham operation group and CLP group. After the experiment, the liver, spleen, lung and kidney of mice were collected for follow-up experiment.

### Isolation of neutrophils

Mice were intraperitoneally injected with 1 mL 10% peptone, and the same dose of peptone was injected 12 h later. Three hours later, the mice were killed and put into 75% alcohol for 5 min. Then put the mice on the super-clean worktable. Mice were intraperitoneally injected with 4 mL RPMI-1640 medium and then gently rubbed their abdomen for 3–5 min to extract the peritoneal fluid. Repeat twice. The neutrophils were isolated after centrifugation at 1500 rpm for 5 min. Refer to the instructions for specific steps (LZS1100, tbdscience, China). Blood drawn from the veins was collected in negative pressure anticoagulant tubes treated with sodium citrate. For specific separation steps, refer to the kit instructions (LZS11131, tbdscience, China).

### Bacterial strains and growth conditions

The *S. aureus* strain (provided by the microbiological and immunological laboratory at Jilin University, USA 300) or *E.* *coli* strain (provided by the pharmacology laboratory at Jilin University, ATCC29522) was grown in brain heart infusion agar (BHI) (HB8478, Hopebio, China) media at 37 ℃ with shaking at 200 rpm. When bacteria were cultured to mid-logarithmic phase of growth, collected precipitates (7000 r/min, 3 min) and diluted with phosphate buffer saline (PBS) media (OD 600 = 1).

### Flow cytometry analysis

To determine the purity of the neutrophils, flow cytometry analysis was performed. Briefly, cells were stained with antibodies against neutrophil surface markers PE Rat Anti-Mouse Ly-6G (551461, Becton, Dickinson and Company, USA) and FITC Rat Anti-CD11b (557396, Becton, Dickinson and Company, USA) for 30 min at 4 ℃. The resulting supernatant was discarded (1000 r/min, 5 min), and cells were washed and resuspended in at least 1 mL of PBS for flow cytometry analysis (Beckman CytoFLEX, USA).

### Quantification of DNA release from activated neutrophils

Fleshly isolated neutrophils were resuspended with RPMI-1640 medium and the cell concentration was adjust to 1 × 10^6^/mL and then seeded 1 × 10^5^/200 µL into 96-well plates and stimulated with *S. aureus* or *E. coli* (MOI = 10, 1 h) or LPS (20 µg/mL, 1 h). Sytox Green (Invitrogen), a non cell-permeant DNA binding dye, was added to the cells at a final concentration of 5 μM to detect extracellular DNA. Non-stimulated neutrophils were used a control. The plates were read in a fluorescence microplate reader (Tecan, Switzerland) with a filter setting of 488 (excitation)/523 (emission).

### NETs formation by stimulation with bacteria or LPS and confocal laser-scanning microscopy

Neutrophils were resuspended with RPMI-1640 medium. 2 × 10^5^ cells were stimulated with *S. aureus* or *E. coli* (MOI = 10, 1 h) or LPS (20 µg/mL, 1 h) on poly-lysine pre-coated cover glass slides after 1 h, respectively. After incubation, the cells were fixed with 4% paraformaldehyde for 20 min at RT, washed three times with PBS, permeabilized with 0.1% Triton X-100 for 20 min, washed three times with PBS and blocked in 5% donkey serum/PBS, followed by incubation with antibodies to histone 3, MPO and NE at 4 ℃ overnight. Anti-histone 3 antibody, anti-MPO antibody and anti-NE antibody were used for the detection of histone 3, MPO and NE in *S. aureus* or *E. coli* or LPS-triggered NETs-like extracellular structures. Then, the sample was incubated with the second conjugated antibody for 1 h at RT, washed three times with PBS and then using DAPI to detect DNA. Confocal microscopy was performed as previously described (AMP-activated protein kinase enhances the phagocytic ability of macrophages and neutrophils.) using a confocal laser-scanning microscope (FV-3000, Lacia, Germany) provided by the High Resolution Imaging Facility at Jilin University.

### Extracellular bacterial killing with intact neutrophils

Experimental conditions included medium alone, medium containing WT/GPR109A^−/−^ neutrophils, medium containing WT/GPR109A^−/−^ neutrophils and *S. aureus* with or without NA, medium containing WT/GPR109A^−/−^ neutrophils and *S. aureus* and cytochalasin B (10 μg/mL) with or without NA, and medium containing WT/GPR109A^−/−^ neutrophils and *S. aureus* and 100 U/mL deoxyribonuclease I (DNase I) (Sigma) with or without NA. All the ratio of neutrophils to bacteria is MOI = 10. Control wells contained bacteria without neutrophils. Experiments were carried out in triplicate and repeated at least thrice. Plates were incubated for 30 min at 37 ℃ in a humidified CO_2_ incubator and thereafter kept on ice for further processing. Plates were centrifuged (400×*g*) for 3 min at 4 ℃ to separate neutrophils from bacteria in the suspension. The suspension (CFU extracellular), extracellular bacteria, was separated and paced on ice before culture. Neutrophil pellets were suspended with PBS containing penicillin–streptomycin liquid to kill any adherent extracellular bacteria and washed twice to remove gentamicin. Neutrophils were lysed with 0.1% Triton X-100 on ice for 20 min to release intracellular bacteria (CFU intracellular). All bacterial samples were serially diluted and plated onto HBI agar to determine the CFU count. The percentage of phagocytosis by neutrophils in wells containing DNase to inhibit NETs formation was determined by using the equation (1 − CFU extracellular/CFU intracellular) × 100. The percentage of intracellular killing by neutrophils in replicated wells containing DNase was determined by using the equation (1 − (CFU intracellular/CFU control − CFU extracellular)) × 100. The percentage of killing by NETs in replicated wells containing cytochalasin B to inhibit phagocytosis was determined by using the equation (1 − CFU extracellular/CFU control) × 100.

### Total RNA extraction and qRT-PCR

Neutrophils were re-suspended in ice-cold Trizol solution. Putting for 10 min at RT then the cells were transferred to 1.5 mL non-enzyme EP tube. Total RNA concentration was measured with an RNA Easy Kit. PCR conditions were as follows: 94 ℃ pre-degeneration for 5 min, and then 94 ℃ for 30 s, 35 cycles of denaturation at 50–60 ℃ for 30 s, at 72 ℃ for 30 s and finally at 72 ℃ for 10 min. Primer specificity was estimated by melting curves. Standard curves were generated with reference cDNA to determine the starting quantity of mRNA in all samples.

### Scanning electron microscopy

Cells were fixed with 0.5% glutaraldehyde, postfixed in cacodylate buffer containing osmium tetraoxide, dehydrated by ethanol, dried, mounted on stubs, and sputter-coated with gold palladium alloy. Since the NETs are fragile, each step was done with minimal disturbance of the media to preserve the structures. The morphology of NETs was imaged under scanning electron microscopy.

### Histopathologic evaluation of tissues

Histological analysis the tissues were fixed in fresh 4% formaldehyde solution for 24 h, then dehydrated, transparent, waxed, and paraffin-embedded. Finally, it was cut, stained with hematoxylin–eosin 5-μm parts. Tissue sections were observed under a light microscope to examine the histopathology of the liver, spleen, lung and kidney.

### Tissue homogenates and MPO assay

One tissues in each group were weighed and homogenized in HEPES, with ice compress ratio of 1:4. After fully grinding, homogenate was transferred to a new suitable centrifuge tube and centrifuged at 13,000 rpm for 20 min. The supernatant was collected into a new centrifuge tube for enzyme-linked immunosorbent assay (ELISA), and the sediment was combined with 0.5% CTAC equal to HEPES and was centrifuged again at 13,000 rpm for 20 min in order to get rid of the remaining lipid. The supernatant was the MPO sample. Each MPO sample (75 μL) and substrate (75 μL), 3,3′,5,5′-Tetramethylbenzidine 3 mM (8798 μL), Resorcinol 6 mM (180 μL) and H_2_O_2_ 3% (2.5 μL) were added to a 96-well plate for 3–5 min. Finally, 100 μL of H_2_SO_4_ (2 M) was added to terminate the reaction. The absorbance peak (OD value) was detected at a wavelength of 450 nm by a microplate reader. The concentration of the analyte was based on the OD value.

#### Enzyme-linked immunosorbent assay

The protein levels of TNF-α, IL-6 and IL-1β in the supernatant of the homogenate were evaluated with the corresponding ELISA kits according to the manufacturer’s instructions (Biolegend, San Diego, CA92121, USA).

#### Western blot analysis

The tissues were lysed in lysis buffer (Beyotime, Shanghai, China). The protein concentrations were measured using the bicinchoninic acid (BCA) protein assay kit (Thermo Fisher Scientific, USA). A total of 30 μg protein was separated by 10% sodium dodecyl sulfate–polyacrylamide gel electrophoresis SDS-PAGE. The separated proteins were subsequently transferred onto a polyvinylidene difluoride (PVDF) membrane (Millipore, Darmstadt, Germany). After blocking with 5% nonfat milk for 2 h at room temperature, the membranes were incubated overnight at 4 °C with primary antibodies against histone H3 (1:2000) (Cell Signaling Technology, Danvers, MA, USA), citrulline histone H3 (1:1000) (abcam, UK), GPR109A (1:500) (Santa Cruz Biotechnology, California, USA) and β-actin (1:8000) (proteintech, Wuhan, China). Subsequently, the membrane was washed five times in 0.05% Tris-buffered saline with Tween-20 (TBST, pH 7.4) for 10 min each time and was then incubated with an HRP-conjugated anti-mouse (1:20,000) or anti-rabbit secondary antibody (1:10,000) (Bosterbio, USA) for 1 h on a shaker at RT. The membrane was again washed five times for 10 min each with TBST. Finally, a SuperEnhanced chemiluminescence detection kit (Applygen Technologies Inc, Beijing, China) was used to visualize the immunoreactive proteins. The protein bands were visualized after exposure of the membranes to X-ray film.

### Bacterial culture test

Fresh tissue samples were weighed and PBS was added to grind it in a 1(g):9 (mL) ratio. After grinding, the supernatant was centrifuged (4 °C, 12,000 rpm for 10 min) and absorbed. The supernatant was diluted 10 times and 50 μL liquid was applied to the pretreated solid BHI medium. Count the colonies on the culture medium after 24 h.

#### Data and statistical analysis

Images and analysis were generated using GraphPad Prism software (La Jolla, CA, USA). Based on our extensive experience with mouse models of LPS and the planned analytical frame-work, we estimated the number of mice per group required to detect effects of interest at *P* < 0.05. All data are presented as the mean ± SD. One-way-ANOVA (general linear model) was used for comparisons of more than two groups. In cases where overall F-tests were significant (*P* < 0.05), post hoc comparisons using Tukey’s method of adjustment were conducted to determine the degree of significant pairwise differences.

## Results

### The formation of NETs in blood of leukemia patients and its correlation with GPR109A

**Fig. 1:**
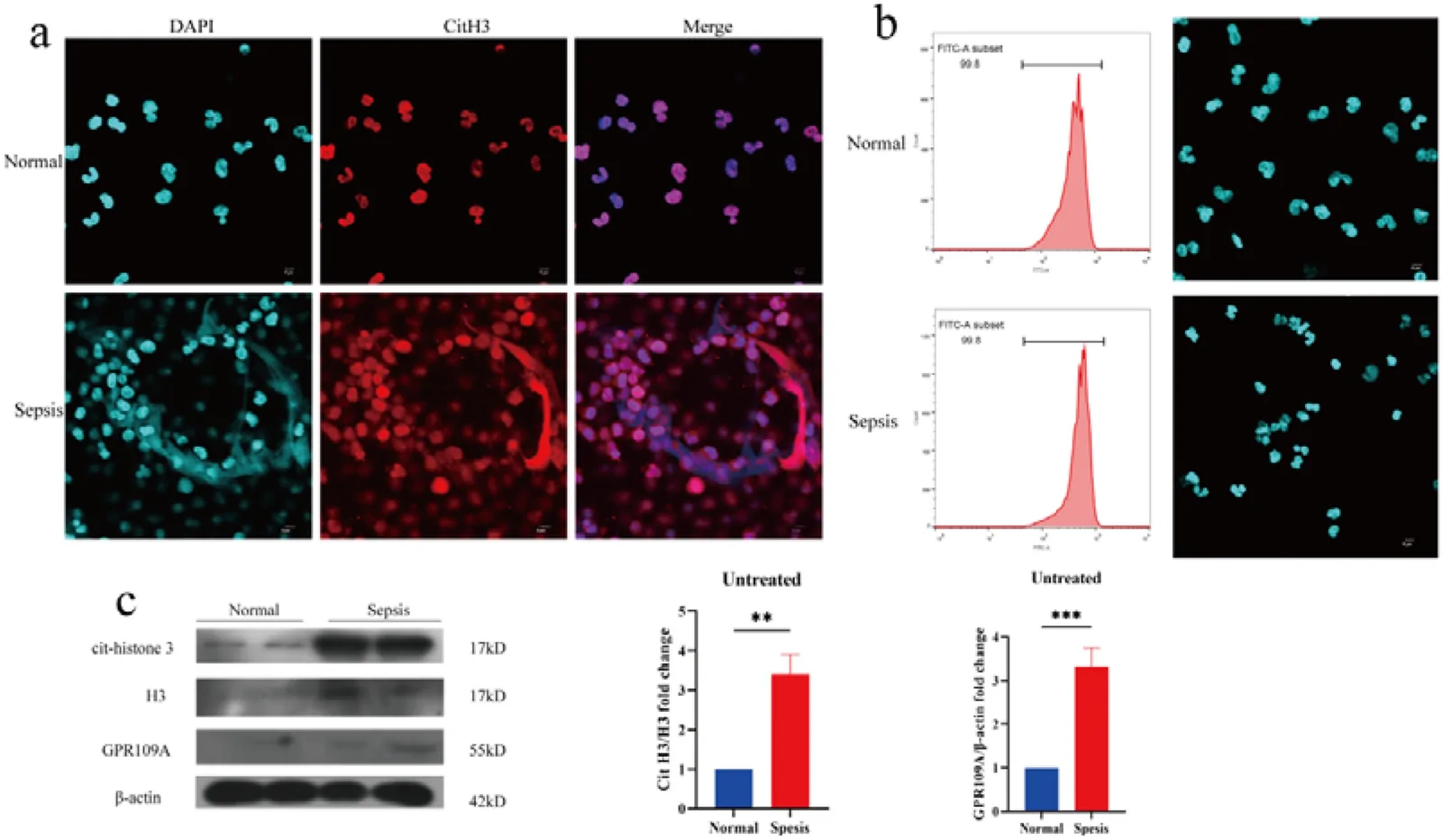
Formation of NETs in patients with sepsis. We isolated neutrophils from the blood of patients with sepsis and healthy people. **a** Immunofluorescence results of human neutrophils stained by CitH3. **b** Flow cytometry detection and immunofluorescence detection of human neutrophils. **c** Protein levels of GPR109A and CitH3 in neutrophils. Values are presented as means ± SEM (n = 5) (*p < 0.05, **p < 0.01, ***p < 0.001, ****p < 0.0001)

NETs will be formed in large quantities after bacteria or their toxins enter the blood [24]. We isolated neutrophils from septicemic patients and healthy people. Flow cytometry results showed that the content of isolated neutrophils was more than 90% (Fig. 1b). After 30 min of culture, neutrophils from septic patients appeared a large number of NETs (Fig. 1a). This suggested that neutrophils in septic patients will form NETs spontaneously. Then we detected the protein levels in neutrophils. The results showed that citrullinated histone and GPR109A were significantly up-regulated in neutrophils of patients with sepsis (Fig. 1c). This suggested that GPR109A may be involved in the formation of NETs.

### Isolation of neutrophils and the effect of GPR109A on the key gene PAD4 of NETs

**Fig. 2:**
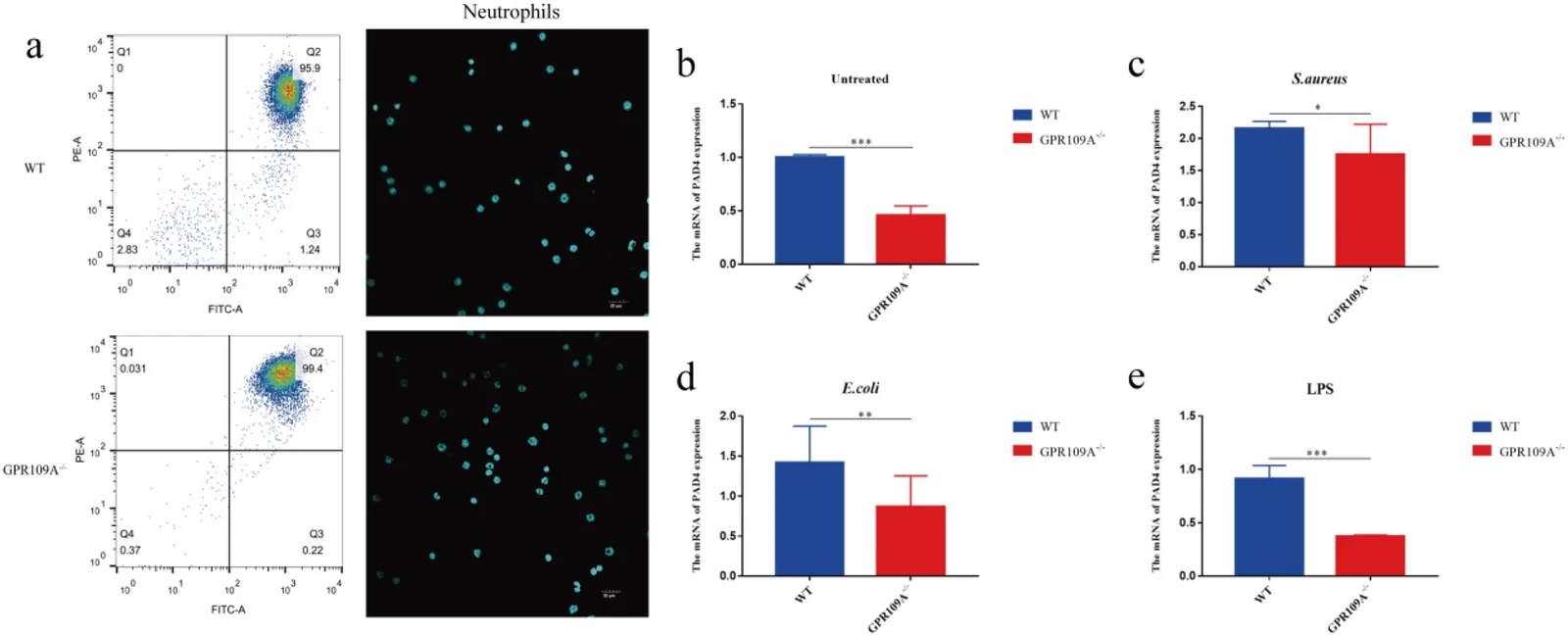
Isolation and identification of neutrophils. We collected neutrophils from mouse abdominal cavity, and then identified them by laser confocal microscopy and flow cytometry. The antibodies used in flow cytometry were Ly-6G and CD11b. **a** Identification results of neutrophils; Confocal pictures of neutrophils. **b**–**e** The gene levels of PAD4 in neutrophils in WT and GPR109A^−/−^ mice. Values are presented as means ± SEM (n = 3) (*p < 0.05, **p < 0.01, ***p < 0.001, ****p < 0.0001)

We isolated neutrophils from GPR109A^−/−^ mice and WT mice with high purity (Fig. 2a). Then we detected the expression of PAD4 in untreated group, *S. aureus* aureus group, *E. coli* group and LPS group. The results showed that the gene expression of PAD4 was significantly down regulated after deficiency of GPR109A (Fig. 2b–e).

### Effects of different time and concentration of GPR109A on NETs

**Fig. 3:**
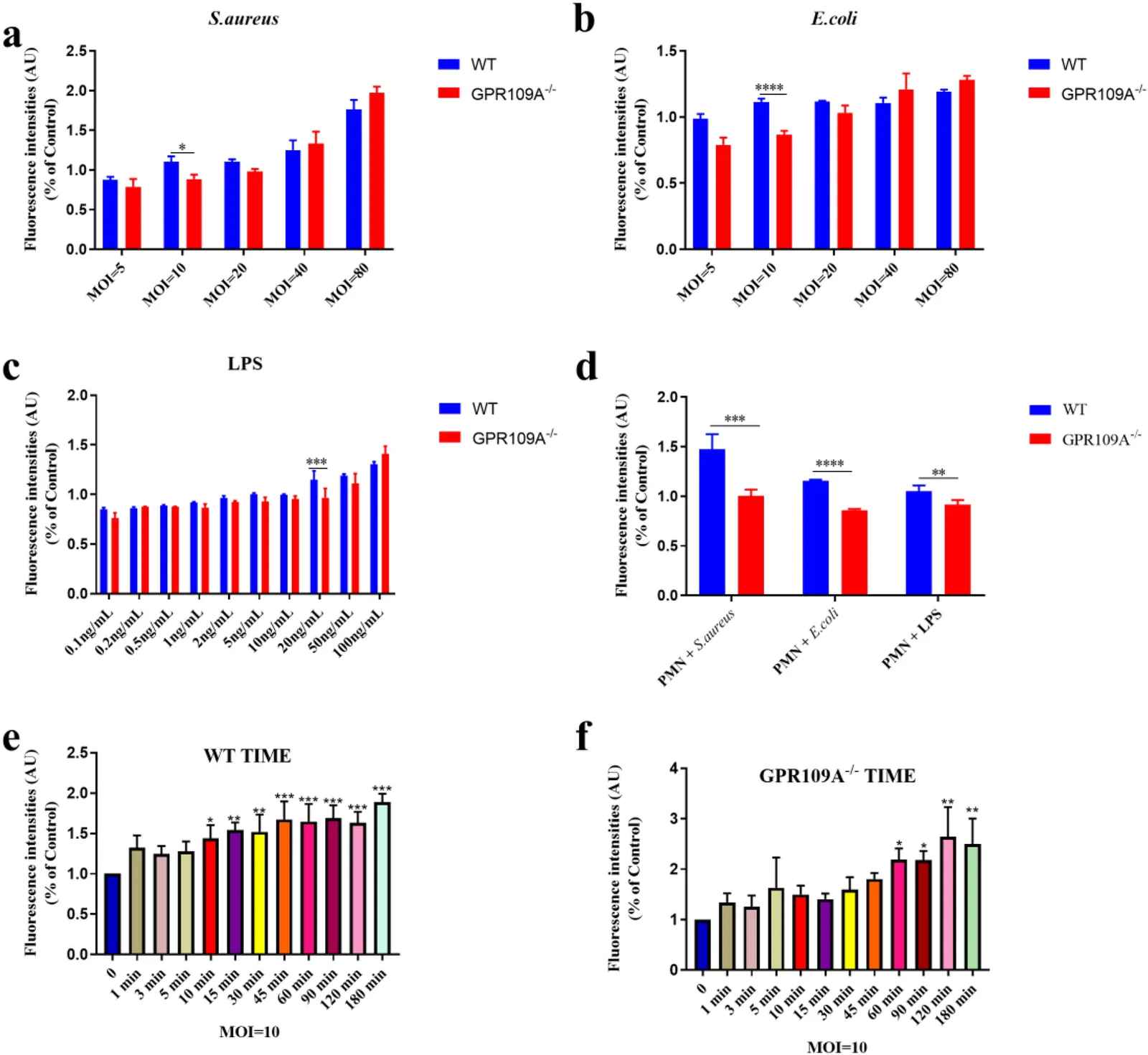
Effect of GPR109A on NETs formation. **a**–**d** We stimulated neutrophils with different concentrations of *E. coli*, *S. aureus* and LPS for 30 min. The results showed that GPR109A had significant difference in the formation of NETs when MOI = 10. **e**–**f** We stimulated neutrophils with *E. coli* with MOI = 10. Effect of GPR109A on NETs formation at different time points. Values are presented as means ± SEM (n = 6) (∗*p* < 0.05, ∗∗*p* < 0.01, ∗∗∗*p* < 0.001, ∗∗∗∗*p* < 0.0001)

Different kinds of bacteria or toxins have different effects on NETs. We stimulated neutrophils in GPR109A^−/−^ and WT mice with *S. aureus*, *E. coli* and LPS, respectively. The results showed that when MOI = 10, NETs forming ability of neutrophils in WT mice increased significantly, while that in GPR109A^−/−^ mice decreased significantly. The above situation will also occur when the LPS concentration is 20 ng/mL. Although the formation of NETs was observed by LPS stimulation, this result may also be due to the interaction between platelets and neutrophils. It has also been reported that activation of TLR4 can lead to the formation of NETs. When excessive bacteria or toxins are produced, NETs forming ability of neutrophils will not be reduced due to the deficiency of GPR109A (Fig. 3a–d). This suggested that GPR109A plays a key role in enhancing the ability of neutrophils to form NETs during early bacterial or toxin invasion. In order to further verify the above conjecture, we detected the formation of NETs in GPR109A^−/−^ mice and WT mice at different times. The results showed that the formation of NETs in neutrophils of WT mice increased significantly at 10 min, while the formation of NETs in neutrophils of GPR109A^−/−^ mice increased significantly at 60 min (Fig. 3e, f).

### Effect of GPR109A on early reticulation of neutrophils

**Fig. 4:**
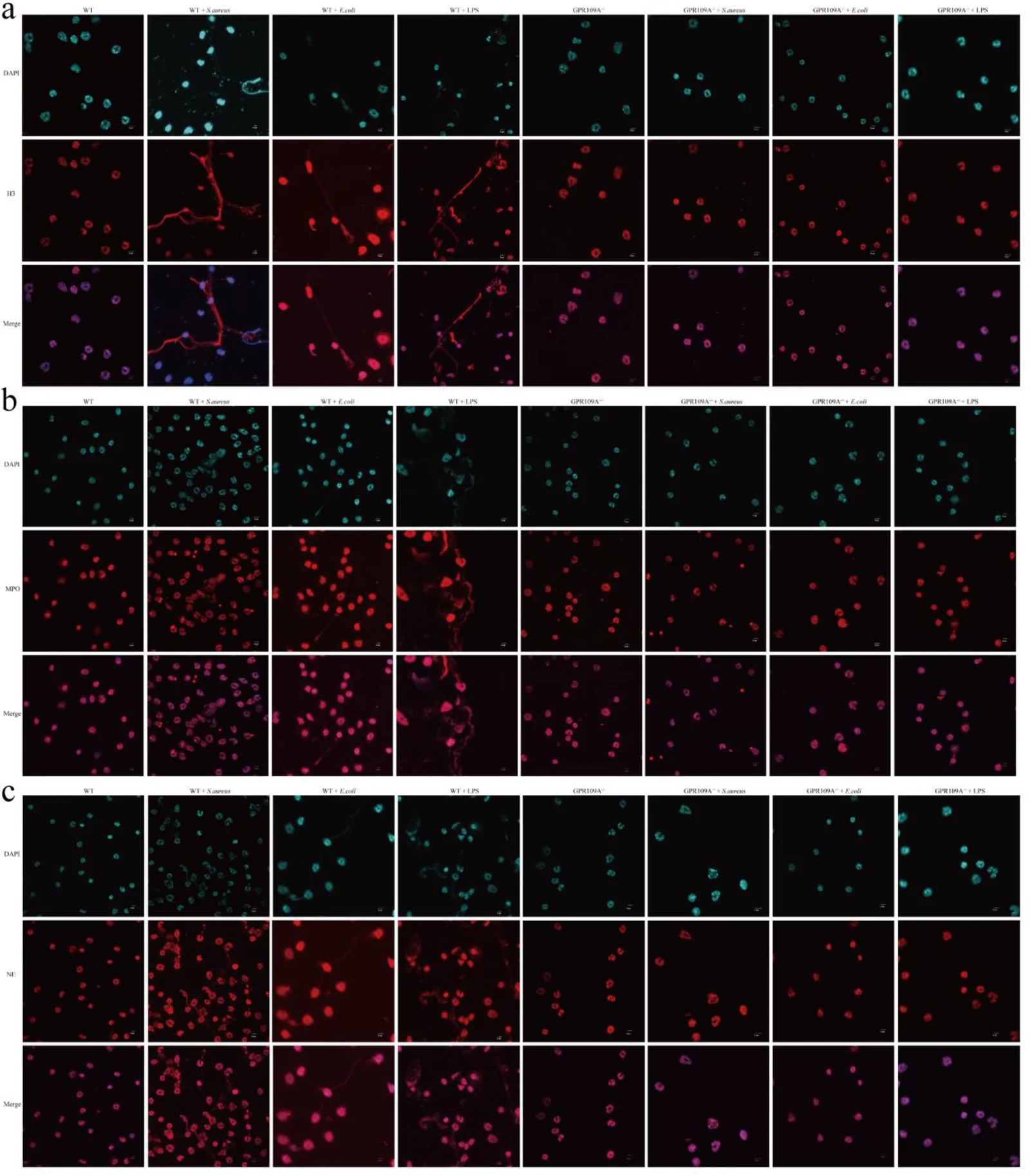
Effects of *E. coli, S. aureus* and LPS on neutrophils in WT and GPR109A^−/−^ mice. Neutrophils from WT and GPR109A^−/−^ mice were stimulated by *E. coli*, *S. aureus* and LPS, respectively. NE, MPO and H3 in NETs were stained by immunofluorescence. **a**–**c** Immunofluorescence staining results of NE, MPO and H3. All results were observed under laser confocal microscope

**Fig. 5:**
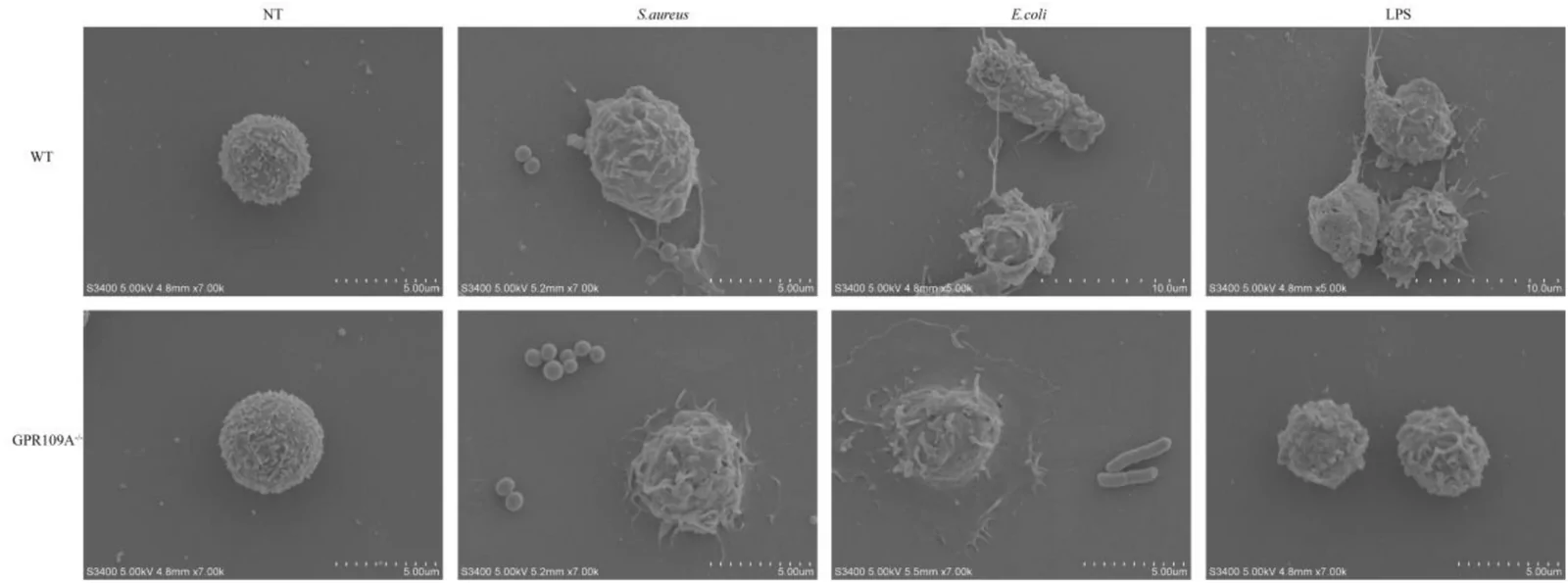
GPR109A knockout reduces the sensitivity of NETs formation. Neutrophils from WT mice and GPR109A^−/−^ mice were isolated and stimulated with *S. aureus, E. coli* and LPS, respectively. The results showed that knockout of GPR109A significantly reduced the sensitivity of NETs formation

Current studies have found some factors that inhibit or promote the formation of NETs [24]. In our study, we found that GPR109A can inhibit NETs formation in the early stage. We stimulated mice neutrophils with *S. aureus*, *E. coli* and LPS for 30 min, respectively. The results showed that the NETs forming ability of neutrophils decreased significantly after knockout of GPR109A. Then, we stained histone 3, MPO and NE, respectively. The results showed that WT mice contained a large amount of MPO, histone 3 and NE, while GPR109A^−/−^ mice rarely formed NETs (Fig. 4a–c). Then we conducted a scanning electron microscope test. The results showed that it was difficult for *S. aureus*, *E. coli* and LPS to induce NETs in mice after GPR109A knockout, while WT mice showed normal NETs forming ability (Fig. 5).

### GPR109A controls the formation of NETs by regulating ROS and histone citrullination

**Fig. 6:**
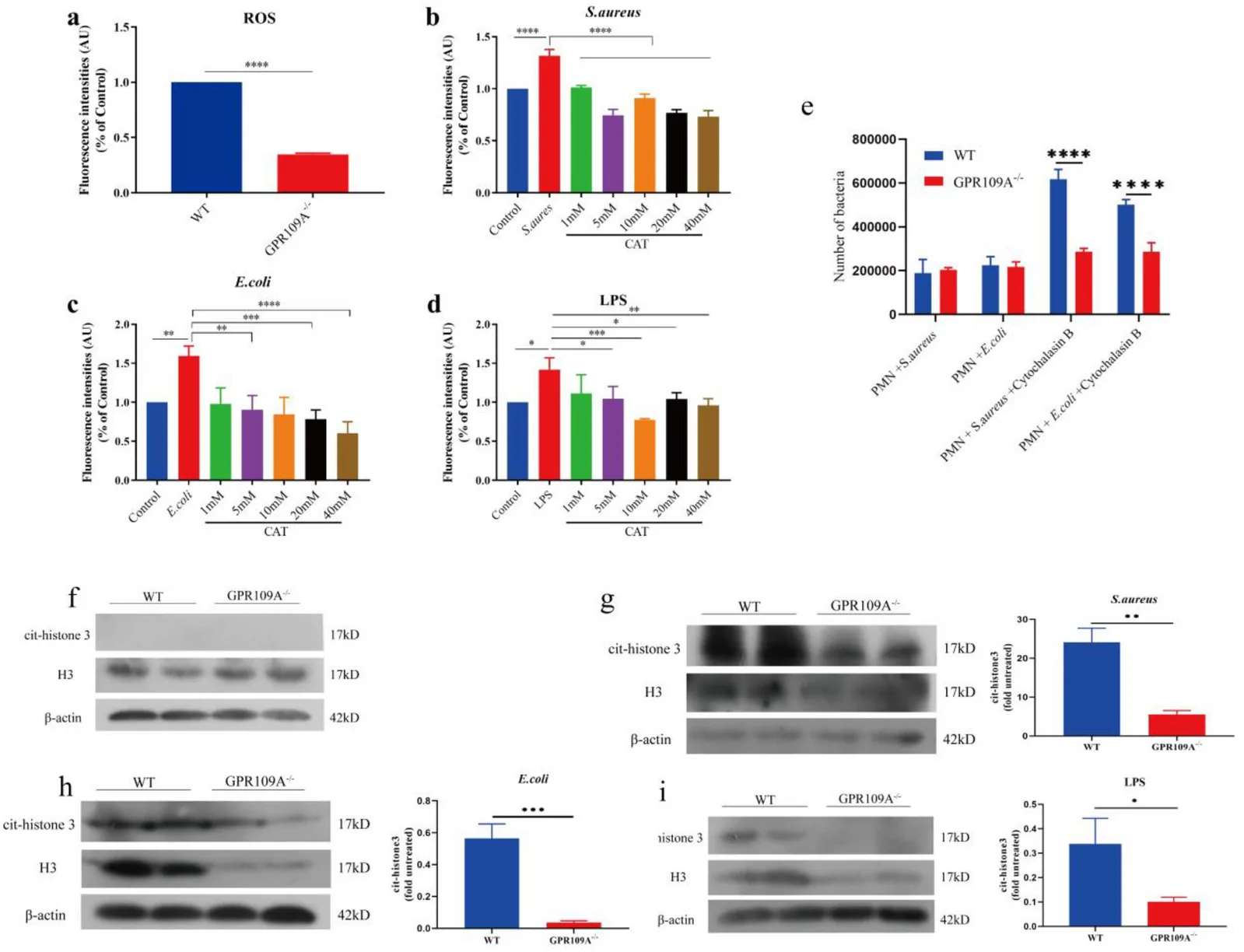
The mechanism of GPR109A on reducing NETs formation sensitivity. **a** Neutrophils were isolated from WT mice and GPR109A^−/−^ mice, and then the ROS level of neutrophils in the two groups was detected. **b**–**d** The NETs formation after inhibition of ROS. **e** The bactericidal ability of NETs. The protein expression in neutrophils was detected by Western blotting. **f** Cit-H3 level in non stimulated mice neutrophils. **g**–**i** Cit-H3 level in neutrophils of WT mice and GPR109A^−/−^ mice stimulated by *S. aureus, E. coli* and LPS. Values are presented as means ± SEM (n = 3) (∗*p* < 0.05, ∗∗*p* < 0.01, ∗∗∗*p* < 0.001, ∗∗∗∗*p* < 0.0001)

Previous studies have shown that ROS and histone citrullination are closely related to the formation of NETs [25]. We found that the level of ROS in neutrophils of GPR109A^−/−^ mice was significantly lower than that of WT mice (Fig. 6a), and the bactericidal ability of NETs decreased significantly after GPR109A knockout (Fig. 6e). In order to further verify the effect of ROS on the formation of NETs, we added ROS inhibitor to neutrophils of WT mice. After stimulation by *S. aureus*, *E. coli* and LPS, we found that inhibition of ROS could significantly reduce the formation of NETs (Fig. 6b–d). Then we detected the histone citrullination of neutrophils in WT and GPR109A^−/−^ mice stimulated by *S. aureus, E. coli* and LPS. The results showed that the citrullination level of histone decreased significantly after GPR109A knockout (Fig. 6f–i). This suggested that GPR109A may control the formation of NETs by affecting histone citrullination.

### Effect of GPR109A on MPO in liver, spleen, lung and kidney in CLP model

**Fig. 7:**
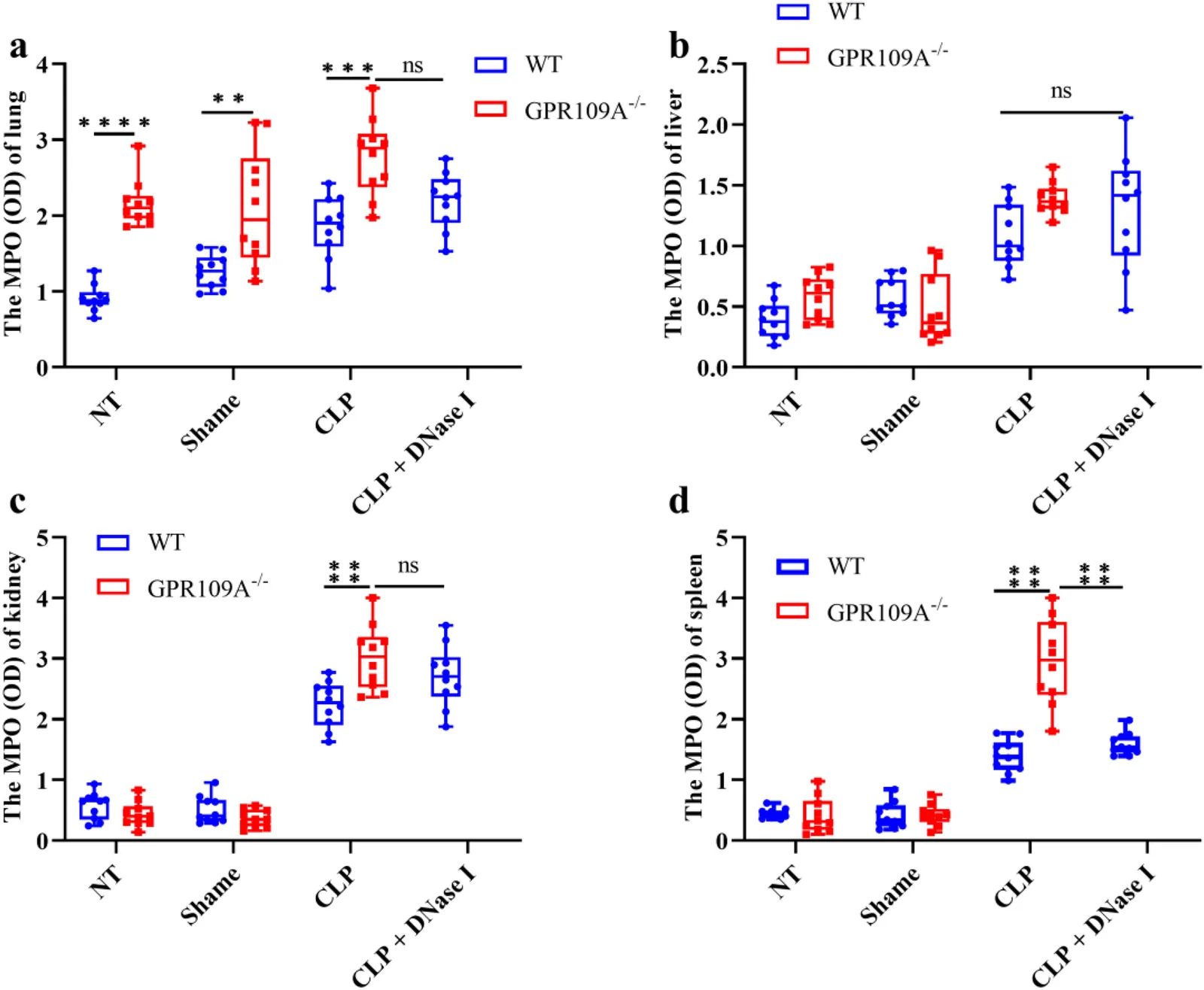
MPO levels of liver, spleen, lung and kidney. We constructed CLP models in WT + CLP, WT + DNaseI + CLP and GPR109A^−/−^ + CLP groups respectively, and collected the liver, spleen, lung and kidney of mice after 3 days. NT means no treatment, Shame means Only surgery was performed without puncture or ligation of the cecum, CLP means cecal puncture and ligation were performed, DNaseI + CLP means Cecal puncture and ligation were performed and DNaseI was injected. **a**–**d** MPO levels of liver, spleen, lung and kidney. Values are presented as means ± SEM (n = 10) (∗*p* < 0.05, ∗∗*p* < 0.01, ∗∗∗*p* < 0.001, ∗∗∗∗*p* < 0.0001)

The invasion of bacteria or toxins into the blood will cause sepsis, and NETs plays an important role in this process [26]. Therefore, we constructed mice cecum ligation and puncture (CLP) model to simulate the occurrence of sepsis. Then, we detected the content of MPO in liver, spleen, lung and kidney of mice. We found that after knockout of GPR109A, the MPO level of each group in lung increased significantly (Fig. 7a). In the liver, there was no significant change in MPO levels between CLP model mice (Fig. 7b). In the kidney, the MPO level of GPR109A^−/−^ mice in CLP model group increased significantly, while that in CLP + DNase I group increased slightly but not significantly (Fig. 7c). In the spleen, the MPO level of GPR109A^−/−^ mice in CLP model was also significantly up-regulated, but there was no significant change in CLP + DNase I group (Fig. 7d).

### Effect of GPR109A on IL-6, TNF-α and IL-1β in liver, spleen, lung and kidney in CLP model

**Fig. 8:**
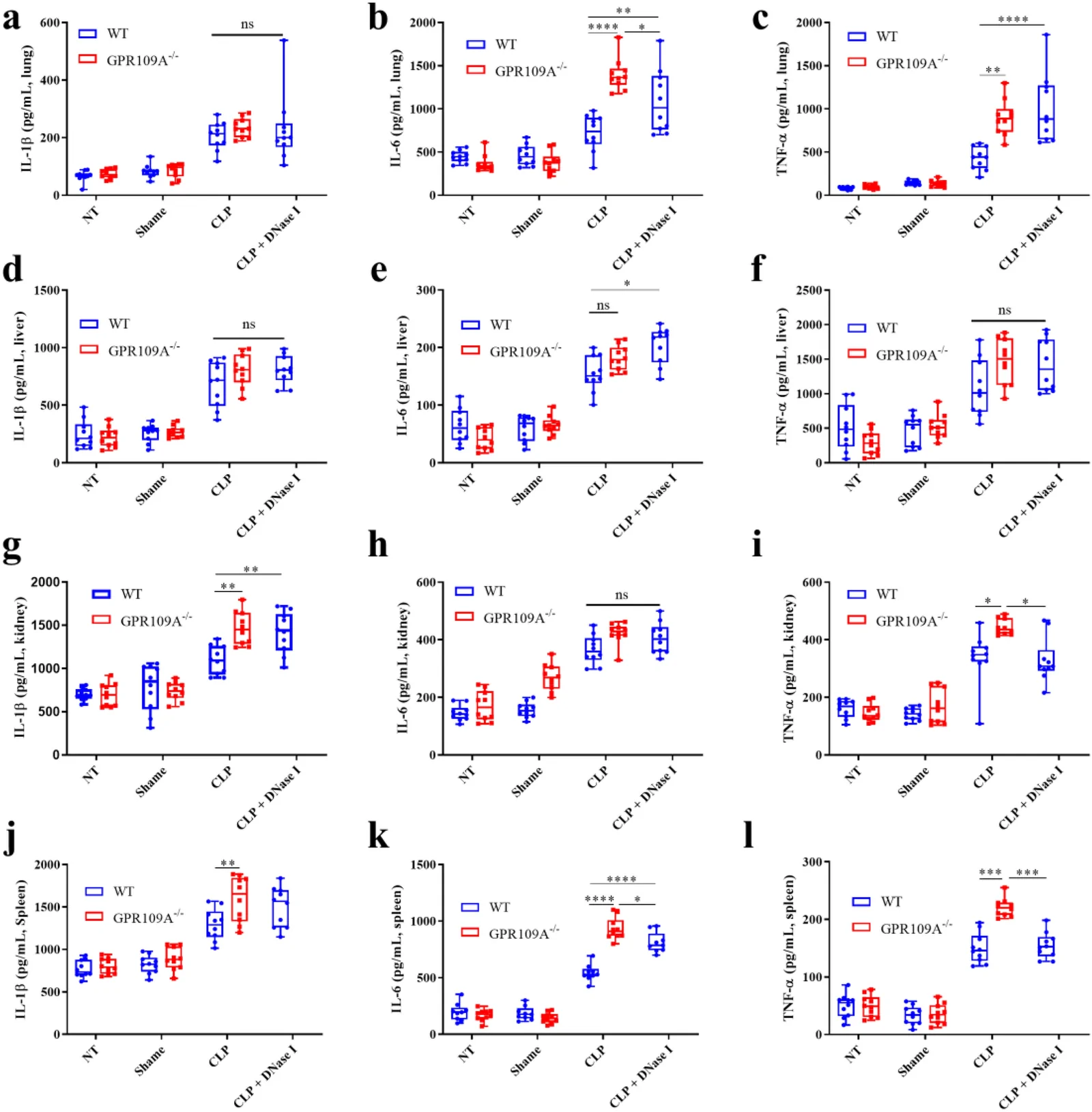
The expression levels of IL-6, TNF-α and IL-1β in liver, spleen, lung and kidney. We constructed CLP models in WT + CLP, WT + DNase I + CLP and GPR109A^−/−^ + CLP groups respectively, and collected the liver, spleen, lung and kidney of mice after 3 days. NT means no treatment, Shame means Only surgery was performed without puncture or ligation of the cecum, CLP means cecal puncture and ligation were performed, DNase I + CLP means Cecal puncture and ligation were performed and DNase I was injected. a–c Expression levels of IL-6, TNF-α and IL-1β in lung. **d**–**f** Expression levels of IL-6, TNF-α and IL-1β in liver. (**g**–**i**) Expression levels of IL-6, TNF-α and IL-1β in kidney. **j**–**l** Expression levels of IL-6, TNF-α and IL-1β in spleen. Values are presented as means ± SEM (n = 10) (∗*p* < 0.05, ∗∗*p* < 0.01, ∗∗∗*p* < 0.001, ∗∗∗∗*p* < 0.0001)

GPR109A can affect the formation of NETs, which plays an important bactericidal role in the early stage of sepsis. In order to further study the effect of GPR109A on the inflammatory response of different tissues in CLP model, we detected the expression of IL-6, TNF-α and IL-1β in liver, spleen, lung and kidney. In the lung of CLP model (Fig. 8a–c), we found that there was no significant difference in the expression of IL-1β among WT group, GPR109A^−/−^ group and DNase I group. However, compared with WT group, the levels of IL-6 and TNF-α in GPR109A^−/−^ group and DNase I group were significantly higher. In the liver of CLP model (Fig. 8d–f), we found IL-1β and TNF-α did not change significantly. Compared with WT group, there was no significant change of IL-6 in GPR109A^−/−^ group, but it was significantly up-regulated in DNase I group. In the kidney of CLP mode (Fig. 8g–i), the content of IL-1β in GPR109A^−/−^ group and DNase I group was significantly higher than that in WT group. However, IL-6 did not change significantly in the three groups. Then we detected the content of TNF-α, the results showed that the content of TNF-α in GPR109A^−/−^ group was significantly higher than that in WT group and DNase I group. In the spleen of CLP model (Fig. 8j–l), the content of IL-1β in GPR109A^−/−^ group was significantly higher than that in WT group. The levels of IL-6 in GPR109A^−/−^ and DNase I groups were significantly higher than those in WT group. The levels of TNF-α in GPR109A^−/−^ was significantly higher than those in WT group, but WT group and Dnase I was no significant.

### Effect of GPR109A on liver, spleen, lung and kidney in CLP model

In order to further understand the effect of GPR109A knockout on CLP model, we stained the tissue sections of liver, spleen, lung and kidney with H&E. In the liver, we found that there were more vacuoles and morphological changes in hepatocytes in WT + CLP + DNase I and GPR109A^−^ + CLP group. The vacuoles in WT + CLP group were smaller and the morphological changes were not significant (Additional file 1: Fig. S2a). In the spleen, we found a large number of immune cell infiltration after CLP modeling (Additional file 1: Fig. S2b). In the lung, we found that the lung wall thickened in varying degrees after CLP modeling, especially in WT + CLP + DNase I and GPR109A^−^ + CLP group (Additional file 1: Fig. S2c). In the kidney, we can obviously observe the congestion and thickening of renal tissue in WT + CLP + DNase I and GPR109A^−^ + CLP group, but only thickening and less congestion in WT + CLP group (Additional file 1: Fig. S2d).

### Effect of NETs on the number of bacteria in blood, liver, spleen, lung and kidney

**Fig. 9:**
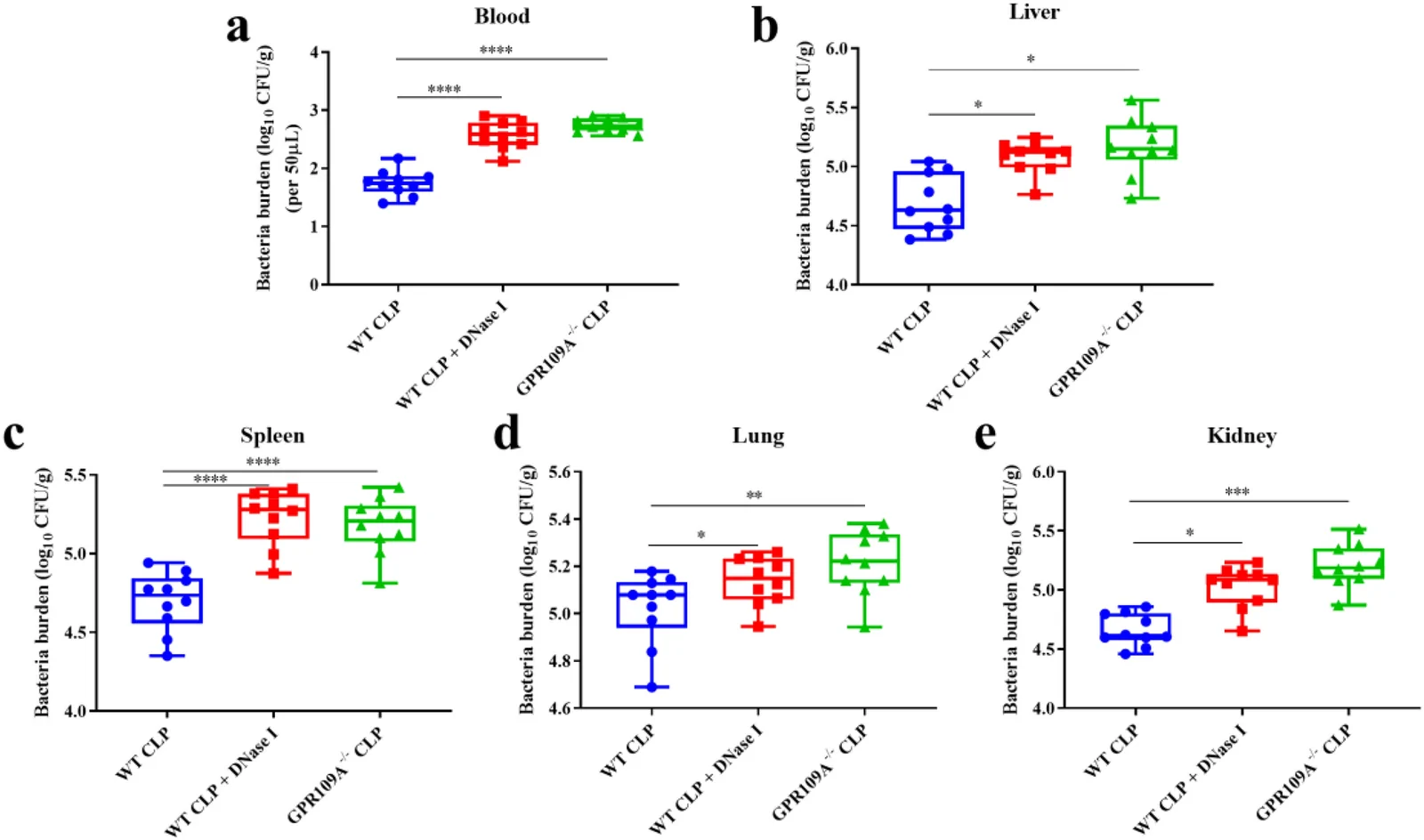
Effects of GPR109A and NETs on the number of bacteria in liver, spleen, lung, kidney and blood. We constructed CLP models in WT + CLP, WT + DNase I + CLP and GPR109A^−/−^ + CLP groups respectively, and collected the liver, spleen, lung, kidney and blood of mice after 3 days. Then the bacteria of blood, liver, spleen, lung and kidney were counted. **a**–**e** The number of bacteria in blood, liver, spleen, lung and kidney. Values are presented as means ± SEM (n = 10) (∗*p* < 0.05, ∗∗*p* < 0.01, ∗∗∗*p* < 0.001, ∗∗∗∗*p* < 0.0001)

Sepsis causes bacteria to enter the blood, and bacteria will enter various tissues and organs with the blood circulation [27]. Bacteria can cause neutrophils in different tissues and organs to form NETs, so as to kill invading bacteria. Firstly, we detected the number of bacteria in blood by spread plate method. The results showed that knockout of GPR109A or inhibition of NETs could lead to the increase of bacteria in blood (Fig. 9a). Then we detected the number of bacteria in liver, spleen, lung and kidney respectively. The results showed that knockout of GPR109A or inhibition of NETs could significantly increase the number of bacteria. At the same time, we found that the number of bacteria in GPR109A^−/−^ CLP group was generally higher than that in WT CLP + DNase I group (Fig. 9b–e).

### The levels of PAD4 and cit-H3 in liver, spleen, lung and kidney

**Fig. 10:**
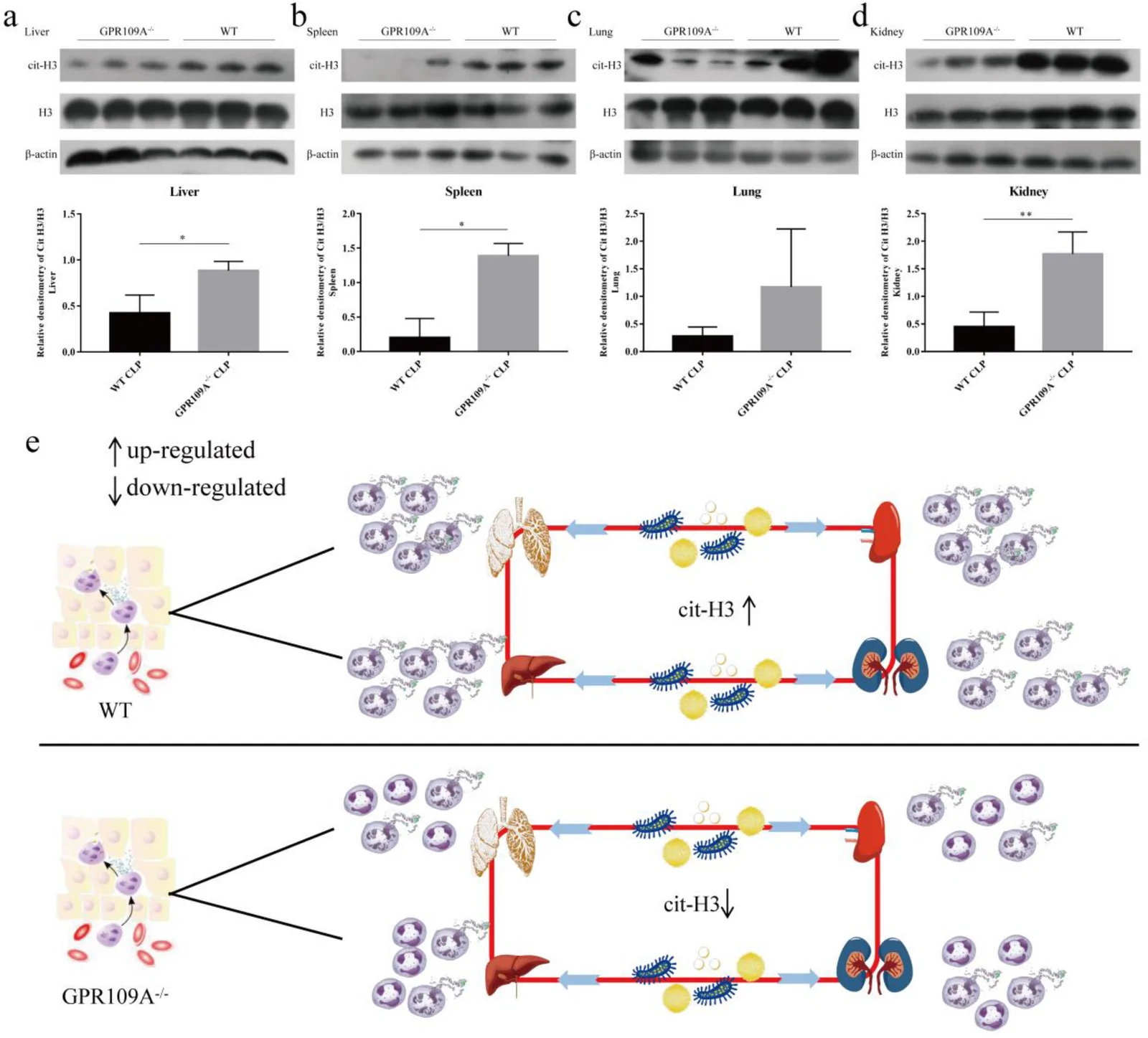
Effect of GPR109A on Cit-H3 in liver, spleen, lung and kidney. The liver, spleen, lung and kidney of WT mice and GPR109A^−/−^ mice in CLP model were collected and extracted the total protein. Then the expression level of Cit-H3 in liver, spleen, lung and kidney was detected by western blotting. **a**–**d** Expression level of Cit-H3 in liver, spleen, lung and kidney. **e** Neutrophils can enter tissues through blood circulation and release NETs in tissues to play a bactericidal function. Cit-H3 is an important marker of NETs formation. Values are presented as means ± SEM (n = 10) (∗p < 0.05, ∗∗p < 0.01, ∗∗∗p < 0.001, ∗∗∗∗p < 0.0001)

Histone citrullination is an important marker of NETs [28]. Our previous studies also showed that the knockout of GPR109A will reduce the levels of PAD4 and cit-H3, thus affecting the formation of NETs. Then we detected the levels of PAD4 and cit-H3 in liver, spleen, lung and kidney. The results showed that the level of PAD4 and cit-H3 in GPR109A^−/−^ group was significantly lower than that in WT group (Fig. 10a–d, Additional file 1: Fig. S1a–d). This result suggested that there may also be relatively few NETs in liver, spleen, lung and kidney in GPR109A^−/−^ group (Fig. 10e).

### Effect of NETs on mortality of CLP model mice

**Fig. 11:**
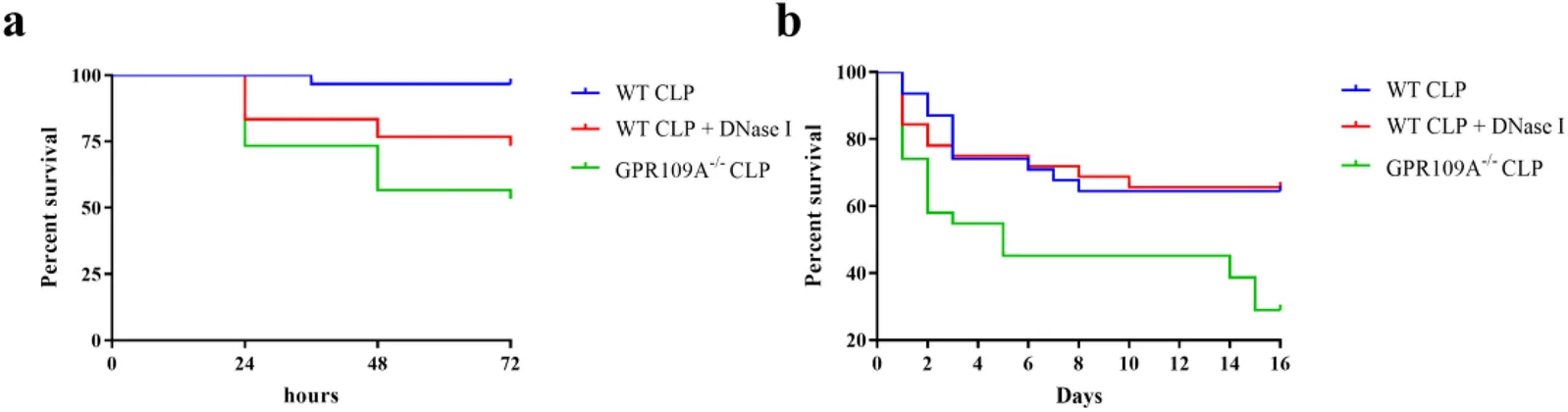
Effects of NETs and GPR109A on mortality of CLP model mice. CLP models were constructed in WT mice, WT + DNaseI mice and GPR109A^−/−^ mice, with 33 mice in each group. DNaseI (100 µL) was injected at a concentration of 100 μg/mL every 2 days. **a**, **b** Mortality of CLP model mice

Acute sepsis is a fatal disease. Therefore, controlling the disease process of sepsis for the first time is of great significance to alleviate acute sepsis [29]. We continuously observed 16 days after the construction of CLP model. The results showed that the mortality of mice in WT CLP group and WT CLP + DNase I group was almost the same on the 16th day, but the mortality of mice in GPR109A^−/−^ group reached 61% (Fig. 11b). However, we also found that when the observation time was 3 days, there was almost no death in WT group, but the mortality of WT CLP group and WT CLP + DNase I group increased sharply. The mortality of WT CLP + DNase I mice was 27%, and that of GPR109A^−/−^ mice was 47% (Fig. 11a). These results suggested that NETs may play an important role in the early stage of sepsis.

## Discussion

**Fig. 12:**
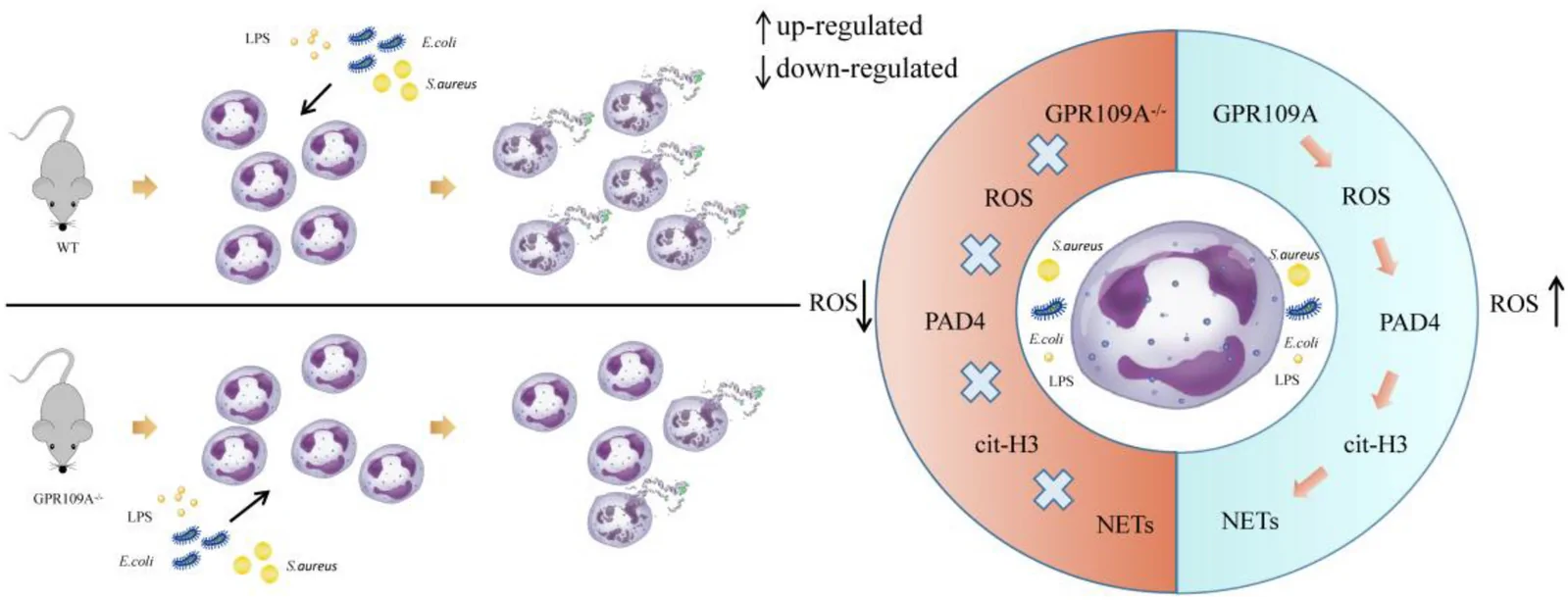
The mechanism of GPR109A on regulating the NETs formation

Sepsis is a critical clinical disease with a very high mortality rate [30]. Neutrophils are the first line of immune defense against sepsis [31]. Our study found that GPR109A is closely related to the early formation of NETs. This may be due to the reduction in ROS levels in neutrophils after GPR109A knockout, which reduces the response of neutrophils to bacteria. Moreover, we also found that knockout of GPR109A significantly increased the mortality of CLP model mice. Inhibition of NETs formation reduces the survival of CLP model mice in the early stage. This suggested that NETs may be related to the early survival of the CLP model. This study proposes a new target for sepsis to helps increase the survival rate of patients with sepsis (Fig. 12).

When bacteria break through the barrier in the body and enter the blood circulation, it leads to severe systemic sepsis [30]. Previous studies have shown that sepsis can cause neutrophils to form NETs and release a large amount of MPO, DNA and other active substances [32]. A study of 55 critical patients in 2018 showed that the circulating level of the MPO-DNA complex in serum increased rapidly and continuously, indicating the early formation of NETs in sepsis [33]. MPO-DNA complex levels were also associated with the severity of organ dysfunction and 28-day mortality [34]. In our study, in isolated neutrophils, we observed a large number of NETs through laser confocal microscopy, which showed that NETs play an important role in the occurrence of sepsis. We also analyzed the protein expression in neutrophils of patients with sepsis. The results showed that GPR109A and cit-H3 were significantly upregulated, which suggested that GPR109A may be closely related to NETs formation.

To further study the relationship between GPR109A and NETs, we extracted neutrophils from GPR109A-knockout mice and WT mice. Previous studies by our research group found that GPR109A can reduce the mortality of sepsis by regulating the intestinal flora [23]. However, the effect of GPR109A-mediated regulation of NETs formation on sepsis has not been reported. We found that GPR109A knockout significantly reduced NETs formation; that is, knockout of GPR109A blocked NETs formation. This result showed that GPR109A knockout may affect neutrophil-mediated killing of exogenous bacteria through NETs in the early stage, prevent neutrophils from responding to exogenous infection in a timely and rapid manner, and increase mortality. Previous studies have shown that ROS are the key factor in killing bacteria and NETs formation by neutrophils [35, 36]. Some studies have also shown that PAD4 can regulate histone citrullination and play a key role in NETs formation [37, 38]. In our study, we found that the expression level of PAD4 was significantly downregulated after GPR109A knockout, and the histone citrullination level in neutrophils, liver, spleen, lung and kidney was also significantly reduced. These results suggested that GPR109A might regulate histone citrullination in multiple organs. Next, we determined the level of ROS in neutrophils. The results showed that the level of ROS was significantly downregulated after GPR109A knockout. Then, we inhibited the production of ROS in neutrophils and detected the NETs formation. The results showed that the NETs formation ability of neutrophils decreased significantly after inhibiting ROS. This further showed that GPR109A mainly changes the sensing of bacteria by neutrophils by regulating the level of ROS.

Studies have shown that NETs can affect the survival rate of patients with sepsis [39]. However, some studies have shown that NETs have strong bactericidal activities and can play an important antibacterial function in the early stage of bacterial invasion into the blood [40, 41]. Therefore, we constructed CLP models in GPR109A-knockout mice and WT mice. Then, we intraperitoneally injected DNase I into some WT mice to eliminate NETs. We measured the MPO levels in the liver, spleen, lung and kidney. The results showed that the MPO levels in the spleen, lung and kidney were significantly upregulated after knockout of GPR109A, but the MPO level in the liver did not change significantly. Then, we analyzed the proinflammatory mediators in the liver, spleen, lung and kidney. The results showed that knockout of GPR109A or addition of DNase I could promote the expression of IL-6, TNF-α and IL-1β. However, we also found that the number of bacteria in the liver, spleen, lung, kidney and blood significantly increased after GPR109A knockout. Previous studies have shown that high levels of proinflammatory mediators may be associated with the enrichment of neutrophils [42]. Under normal circumstances, a high inflammatory level is of positive significance for killing bacteria [43]. Our study found that GPR109A dose not affect the enrichment function of neutrophils, but it weakens the sensitivity of neutrophils to bacteria and reduces the bactericidal activity of neutrophils, including the bactericidal function of NETs. Then, we analyzed the level of histone citrullination in these tissues. The results showed that the NETs formation in the liver, spleen, lung and kidney significantly decreased after GPR109A knockout. This finding also confirms our previous research that knockout of GPR109A will significantly reduces the sensing of bacteria by neutrophils, thus reducing NETs formation.

## Conclusion

Based on the above research, we determined mortality of mice in CLP model mice in the GPR109A^−/−^ and CLP + DNase I groups. Interestingly, compared with WT mice, we found that the mortality of GPR109A-knockout mice continued to increase throughout the observation time of 16 days, while the mortality increased only in the first 3 days after NETs inhibition. There was no significant difference between the WT group and CLP + DNase I group in mortality at 4–16 days. These results showed that NETs were of great significance for the survival of patients with early sepsis, and GPR109A played a regulatory role in the whole occurrence and development of sepsis. Therefore, GPR109A and NETs might be a potential therapeutic targets for treating sepsis and improving the survival rate of patients with sepsis.

## Supplementary Information

**Additional file 1: Fig. S1**. The expression of PAD4 in liver, spleen, lung and kidney. In this experiment, the liver, spleen, lung and kidney of mice were collected, the total RNA was extracted, and then the mRNA levels of PAD4 in liver, spleen, lung and kidney was detected. (a-d) The gene levels of PAD4 in liver, spleen, lung and kidney. Values are presented as means ± SEM (n = 10) (∗p＜0.05, ∗∗p＜0.01, ∗∗∗p＜0.001, ∗∗∗∗p＜0.0001). **Fig. S2**. H&E results of liver, spleen, lung and kidney in CLP model mice. In this experiment, the liver, spleen, lung and kidney of mice were collected. The above tissues were fixed, dehydrated and sliced by formaldehyde and other reagents, and then the liver, spleen, lung and kidney were stained with H&E. (a-d) H&E staining of liver, spleen, lung and kidney. **Fig. S3**. Original WB image of Fig. 1C.

## Data Availability

The materials and methods in the experiment can be requested by the corresponding author or the first author.

## Abbreviations

NETs: Neutrophil extracellular traps
NE: Neutrophil elastase
MPO: Myeloperoxidase
LPS: Lipopolysaccharide
PMA: Phorbol myristate
ROS: Reactive oxygen species
CLP: Cecum ligation and puncture
BCA: Bicinchoninic acid
PVDF: Polyvinylidene difluoride
