# Correction: GPR109A controls neutrophil extracellular traps formation and improve early sepsis by regulating ROS/PAD4/Cit-H3 signal axis

**DOI:** 10.1186/s40164-026-00845-6

**Published:** 2026-10-26

**Authors:** Wenjin Guo, Qian Gong, Xiaofeng Zong, Dianjun Wu, Yuhang Li, Huijie Xiao, Jie Song, Sheng Zhang, Shoupeng Fu, Zhichun Feng, Lu Zhuang

**Affiliations:** 1 College of Veterinary Medicine, Jilin University, Changchun, 130062 China; 2 Senior Department of Pediatrics, The Seventh Medical Center of PLA General Hospital, Beijing, China; 3 Institute of Pediatrics, The Seventh Medical Center of PLA General Hospital, Beijing, China; 4 National Engineering Laboratory for Birth Defects Prevention and Control of Key Technology, Beijing, China; 5 Beijing Key Laboratory of Pediatric Organ Failure, Beijing, China; 6 Department of Gastrointestinal and Colorectal Surgery, China-Japan Union, Hospital of Jilin University, Changchun, 130033 China; 7 College of Animal Sciences, Qian Gong, Zhejiang University, Hangzhou, 310030 China

**Correction: Experimental Hematology & Oncology (2023) 12:15** **https://doi.org/10.1186/s40164-023-00376-4**

After online publication of the article [1] the authors have identified an error in the published Supplementary Fig. 2. During the assembly of the final files, an incorrect image was inadvertently selected from the working folder and published in place of the intended figure. This unintentional oversight was unfortunately missed during final proofing checks. The corrected Supplementary Fig. 2 is provided below. The full and corrected version of the supplementary figure is available with this erratum.

Incorrect Supplementary figure published

**Figure:**
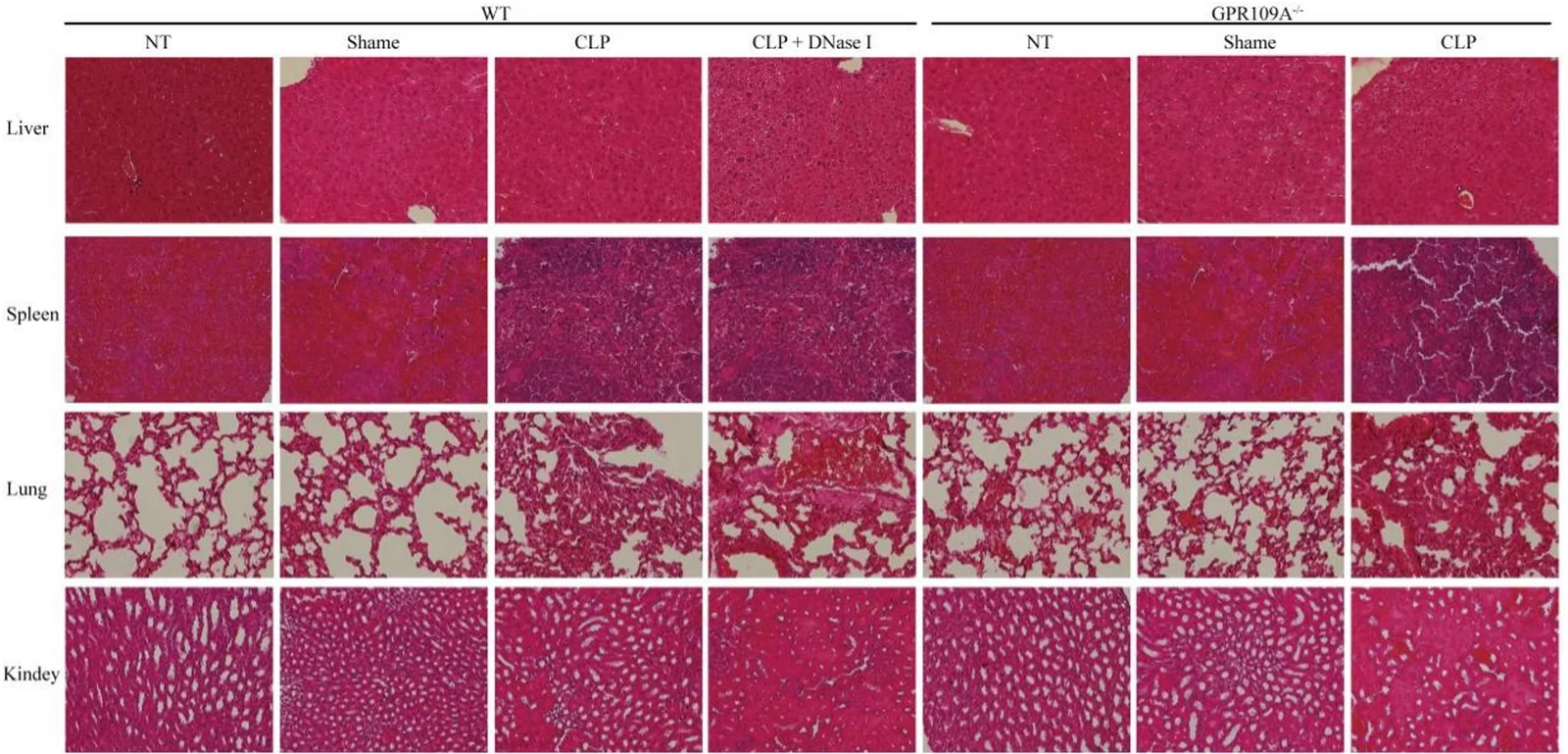

Correct Supplementary figure

**Figure:**
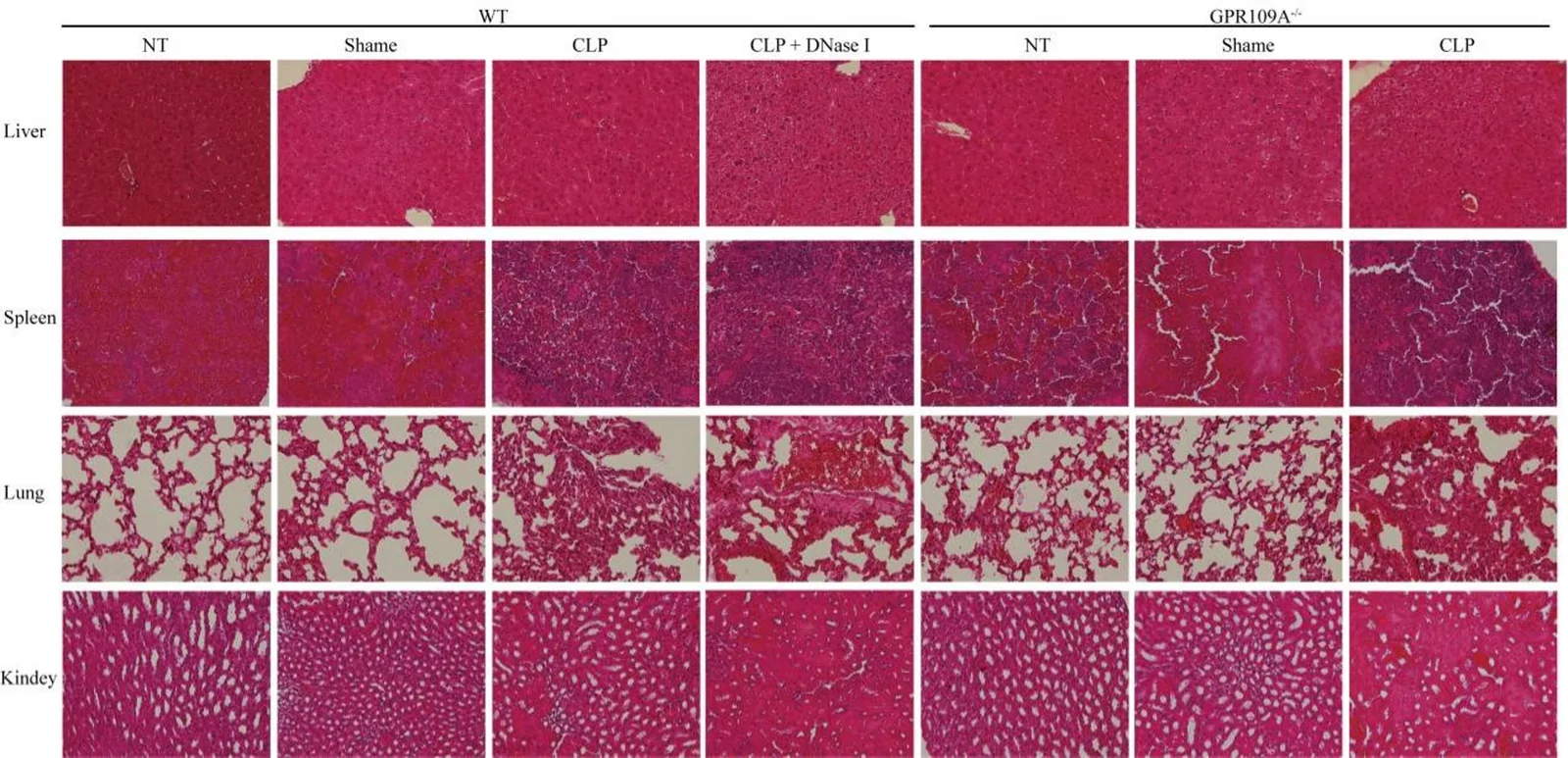

Supplementary Fig. 2. H&E results of liver, spleen, lung and kidney in CLP model mice. In this experiment, the liver, spleen, lung and kidney of mice were collected. The above tissues were fixed, dehydrated and sliced by formaldehyde and other reagents, and then the liver, spleen, lung and kidney were stained with H&E. (a-d) H&E staining of liver, spleen, lung and kidney.

The authors have since re-examined the original raw data and laboratory records to retrieve the correct source file. This error pertains solely to the illustrative image. The corresponding quantitative data were derived from independent experiments and are unaffected. The results, interpretations, and overall conclusions of the article remain entirely unchanged.

## Supplementary Information

Below is the link to the electronic supplementary material.

Supplementary Material 1.
