# Supplementary material for: Correction: GPR109A controls neutrophil extracellular traps formation and improve early sepsis by regulating ROS/PAD4/Cit-H3 signal axis
Source: Exp Hematol Oncol. 2026 Oct 26;15:113. doi: 10.1186/s40164-026-00845-6 (PMC13711891; doi:10.1186/s40164-026-00845-6)
Supplement: Supplementary file 1 [file 40164_2026_845_MOESM1_ESM.docx]

d

c

b

a

Fig. S1. The expression of PAD4 in liver, spleen, lung and kidney. In this experiment, the liver, spleen, lung and kidney of mice were collected, the total RNA was extracted, and then the mRNA levels of PAD4 in liver, spleen, lung and kidney was detected. (a-d) The gene levels of PAD4 in liver, spleen, lung and kidney. Values are presented as means ± SEM (n = 10) (∗p＜0.05, ∗∗p＜0.01, ∗∗∗p＜0.001, ∗∗∗∗p＜0.0001).

d

a

c

b

Fig. S2. H＆E results of liver, spleen, lung and kidney in CLP model mice. In this experiment, the liver, spleen, lung and kidney of mice were collected. The above tissues were fixed, dehydrated and sliced by formaldehyde and other reagents, and then the liver, spleen, lung and kidney were stained with H＆E. (a-d) H＆E staining of liver, spleen, lung and kidney.

Fig. S3. Original WB image of Figure 1C.
